# German dairy farmers’ implementation of veterinary recommendations to improve calf health—a qualitative study based on the transtheoretical model

**DOI:** 10.3389/fvets.2025.1695330

**Published:** 2025-12-19

**Authors:** Marieke Douay--Ryckelynck, Patricia Retzler, Kim K. Meier, Roswitha Merle, Annegret Stock, Katharina Charlotte Jensen

**Affiliations:** 1 Institute of Veterinary Epidemiology and Biostatistics, School of Veterinary Medicine, Freie Universität Berlin, Berlin, Germany; 2 WG Internal Medicine and Surgery, Division Ruminants and Camelids, Farm Animal Clinic, School of Veterinary Medicine, Freie Universität Berlin, Berlin, Germany

**Keywords:** motivational interviewing, veterinary herd health management, implementation of advice, behavior change, calf care workers, implementation rate

## Abstract

**Introduction:**

Calf health is still giving cause for concern, even though best management practices have been known for a long time. This qualitative study aimed to gain insights into the adoption of veterinary recommendations by farmers of large dairy herds to improve the health of calves in Saxony, Germany.

**Methods:**

In the first year of study, nine large dairy farms were visited twice to assess data on calves’ health after assessing the perception of farmers regarding major calves’ diseases. Then, farmers and study veterinarians discussed the results and agreed on three to five measures per farm. Stages of change according to the transtheoretical model, as well as barriers and motivators, were assessed for the following year.

**Results:**

The perception of farmers and the prevalence assessed by study veterinarians correlated moderately. However, the farmers assessed calves’ health better than the comparison with reference data indicated. In total, farmers implemented 15 of 36 recommendations within 1 year (42%). Barriers hindering the implementation were related to concerns that the team would or could not comply, the belief in the effectiveness of measures as well as the old buildings. Recommendations that needed constant changes in management were less likely to be implemented. Especially offering roughage and water to the calves—a measure mostly suggested by the study team—were seldom implemented or were given up before the study ended. However, factors mentioned positively were necessity and simplicity of recommendations.

**Discussion:**

This study indicates that farmers are, in general, willing to adopt measures to improve the health of calves. However, staff shortage and the motivation of team members played a crucial role in these large dairy farms. Moreover, constant feedback and evaluation of success are needed to encourage farmers to maintain those measures that need constant action.

## 1 Introduction

Calf health forms the basis of a successful dairy farm. Many studies conducted in Germany and other countries have shown that there is still room for improvement concerning calf health (1, 2). A large cross-sectional study in Germany (“PraeRi”-study) demonstrated that the mean calf mortality rates from 3 days of life until weaning range from 3.7 to 7.4% (3). In the United States, calf mortality following the same definition was reported to be at 5.0% (4). The most common cause of calf mortality is neonatal calf diarrhea, while bovine respiratory disease (BRD) is considered the second most common cause (5). In the PraeRi-study, high prevalence of calf diseases was observed [omphalitis 21%, diarrhea 19% and respiratory disease 9% (6)].

For more than 40 years, the various reasons underlying calf diseases have been well-known (5, 7). Risk factors for increased calf mortality that were identified were deficits in colostrum management, hygienic conditions in the calving area, calf nutrition, climate control, and animal handling procedures (8–10). Advisory activities of veterinarians and consultants, addressing weaknesses in the calf rearing management, however, did not lead to the desired improvement in calf health on the majority of dairy farms (11–14). For example, it has been recommended on multiple occasions to better monitor colostrum feeding protocols to increase the passive transfer of immunity, but the goals set by experts were met by only a minority of farms (15, 16). Since farm workers are the first in line to address the well-being and fitness issues of the animals (17), their technical knowledge and motivation are essential. Santman-Berends et al. (7) demonstrated that calf mortality is related to priorities, time management, and the mindset of farmers and their employees. In the latter study, farmers either were not aware of high calf mortality on their farm, or felt powerless to find a solution, or assigned higher priorities to other matters than calf rearing (7). Furthermore, communication between the different actors of the farming industry is essential to find and implement solutions, but also to engage in a discussion that would benefit not only the calves but the farmers themselves. Even though the public opinion is pressing for greater animal welfare (18, 19), the human factor that has been neglected in the past, needs more consideration and ideally including the view of social scientists (20). Understanding and influencing farmers’ behavior requires a research approach that captures the complexity of individual motivations, contextual constraints, and social dynamics. Qualitative social research is particularly well-suited for this purpose, as it allows for in-depth exploration of perspectives, meanings, and decision-making processes within their real-life context (21, 22). Unlike standardized quantitative methods, qualitative approaches emphasize openness, reflexivity, and adaptability during data collection, which is essential when investigating heterogeneous and evolving phenomena such as behavioral change on farms (23, 24).

**Figure 1:**
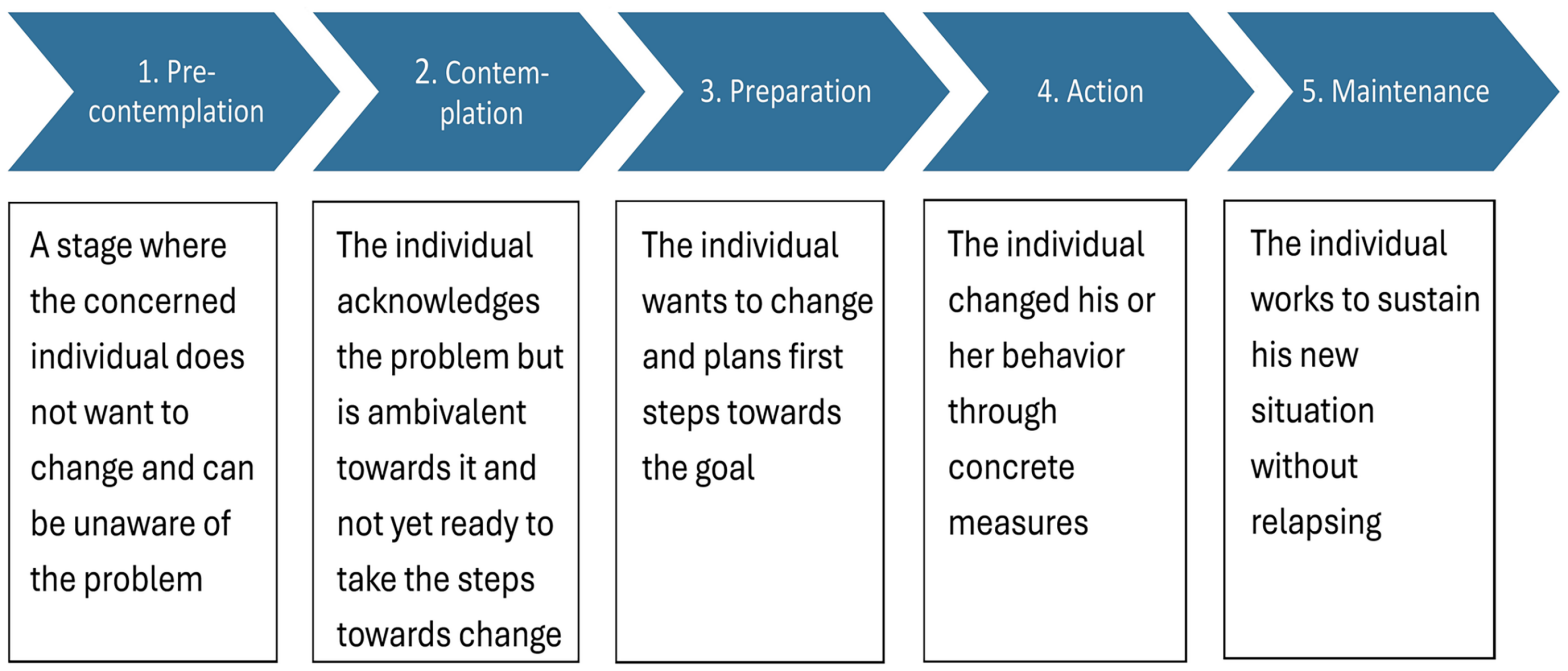
The transtheoretical model as described by Prochaska et al. (26).

In the process of continuous improvement of calf rearing, it is necessary to identify strengths and weaknesses in the daily work routines and the environment in order to reduce the risks of calf mortality. To achieve better calf health, it is necessary—especially on large dairy farms—to change routines in the management of calves and implement new behavior in staff. The transtheoretical model (TTM) was developed by James O. Prochaska, Carlo Di Clemente, and colleagues in the 1970s in order to understand the behavior change process in psychotherapy (25). The approach uses a system of stages to assess the readiness of an individual to change a health habit (26) and subsequently to evaluate the progress in the change process. Originally developed for psychotherapy, it has successfully been applied to the adoption of management measures in agriculture (27, 28). Concerning the husbandry of livestock, only a few studies were grounded on the TTM up to now (29, 30). None of these studies focused on the improvement of dairy calf health. Often measures to improve calf health need a constant behavior change, like changing hygiene routines, and are therefore requiring considerable and durable effort. Therefore, the application of the TTM model on the implementation of those measures is beneficial, as it allows the monitoring of attitude changes on an extended study time. For this study, we used the commonly known stages of the model described in Figure 1.

A lot of studies highlight the strong importance of decisional balance and the TTM (31, 31). The decisional balance describes an individual’s assessment of the perceived advantages and disadvantages of behavioral change and is a key factor in the progression through the stages of change. Marshall et al. (67), Eeckhout et al. (31) as well as Planchard et al. (32) found that the individuals in the early stages of change, especially the preparation, the perceived benefits (motivators) and costs (barriers) of change are relatively balanced, and that the balance between these views or judgments shifted along the stages of change. Therefore, it is not only relevant to assess the stage of behavior change but also to elaborate on the barriers and motivators along the process. Thereby, factors can be identified that might ease the way into a favored behavior, like an improved colostrum management.

Within a longitudinal study in Saxony, Germany, we advised nine farmers or herd managers how to improve calf health on their farms. In an exploratory approach, we assessed the implementation of measures as well as the barriers and motivators to answer the following questions:

- How do the farmers estimate the health of their calves at the beginning of the study? Is it in accordance with the data gathered and the observations made by the study veterinarians?
- Which stage of behavior change concerning the management measures are the farmers in? How do these stages change over time?
- How many measures were successfully implemented? Which measures were easy to implement, and which measures were not implemented?
- What did the farmers initially mention as barriers and motivators? Did the barriers and motivators stay the same as expected, as the efforts of implementation went on?

This study follows an exploratory, qualitative research approach, aiming to gain in-depth insights into how farmers on large-scale dairy farms perceive and respond to veterinary recommendations concerning calf health. Rather than striving for statistical generalizability, the objective was to understand real-life interactions and decision-making processes in a specific context in order to identify practical levers for improving advisory strategies. Qualitative research places emphasis on contextual relevance, meaning-making, and the subjective perspectives of participants, which are essential when examining complex behavioral patterns (21). The findings are thus intended not to represent all dairy farms, but to inform and inspire more targeted and participatory approaches to veterinary communication and implementation support. By answering these questions, we aim to better understand how we can guide farmers in the transition toward better calf care and husbandry with the goal of reducing calf mortality as well as morbidity.

## 2 Materials and methods

### 2.1 Study design

**Figure 2:**
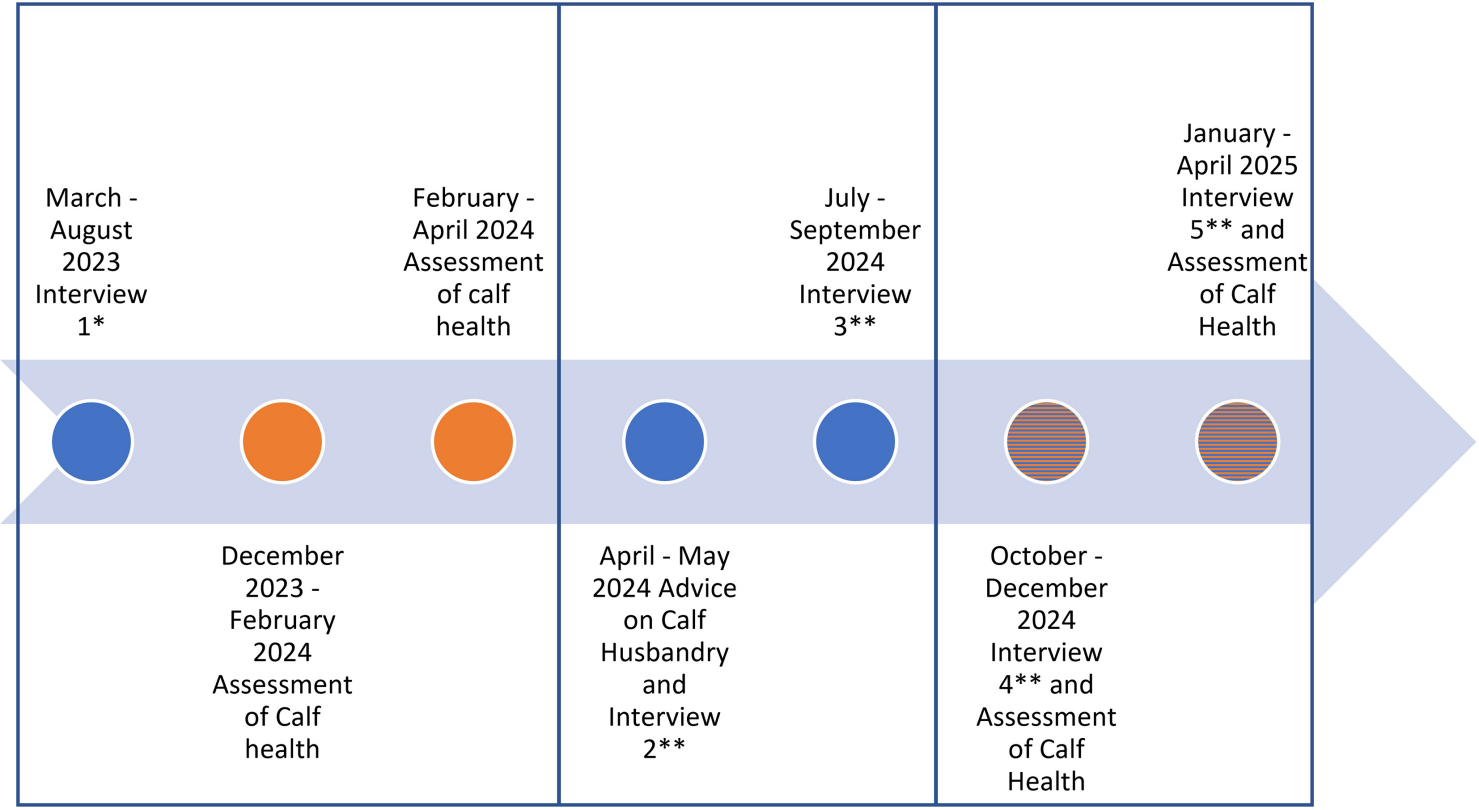
Phases of the study. * General questionnaire, ** Transtheoretical model question form with measures to implement, in orange: assessment of calf health, in blue: interviews.

This study was conducted from April 2023 to April 2025 and was divided into three phases (assessment I, consultation, assessment II; Figure 2): in the first phase, an interview was conducted with the managing director or the herd manager (referred to as farmer in the following). Information on the calving management and health, on the general management of the farm, and on the perception concerning calf health was gathered. Moreover, each farm was visited twice to monitor the health status of the calves by clinical examination and collect information on housing conditions and management. Calves from the second day of life until weaning were included. The visits took place in the cold season 2023/24. At the beginning of the second phase, farms were visited again to present and discuss the results of the first two visits with the managing director. Subsequently, veterinarians made proposals for changes in the calf-rearing process. Finally, consensus on the intended implementation of distinct measures was reached. An additional visit was made to accompany the transition process. In the third phase, each farm was visited twice to collect data on the health status of the calves. In addition, farmers were asked to what extent the measures as agreed upon were implemented in the cold season 2024/25.

### 2.2 Recruitment of participants

An information form with details of the study and application forms for the project was distributed at an advanced training course for farmers in November 2022 in Saxony. Furthermore, an email with the same document was sent from the Saxon State Office for Environment, Agriculture and Geology (Sächsisches Landesamt für Umwelt, Landwirtschaft und Geologie) to their registered farms, and a request was made to the Saxon Animal Disease Fund (Sächsische Tierseuchenkasse) to spread the forms as well.

Inclusion of at least five and at a maximum of 10 farms with more than 500 calvings per year that were willing to participate in the study in order to improve calf rearing was strived for. Inclusion criteria were the willingness to cooperate and to provide access to treatment and performance data of the farm over a period of 2–3 years. Later on, it became clear that the motivation to participate varied between the farms.

The article from Gotti et al. (22) presents a good characterization of the farms selected for this study through the “pattern 5” with the specifics: “expanded workforce, robots and/or sensors, rigorous health-hygiene and biosecurity protocols.” With the expected size of the herd as an inclusion criteria, it is expected that the farm would employ external personnel, have a high level of biosecurity for historical reasons, and not allow grazing, which can be comparable to the results achieved by Gotti et al. (22).

Ten dairy farms applied, of which eight were selected at the start of the project. Two farms did not meet the inclusion criteria. A ninth dairy farm was included at a later stage in the study, and was only surveyed once in the first phase, but then followed the normal course of the study.

Although this last farm entered the project later, all selected respondents participated in every phase of the study. Some managerial decisions were made in the farms during the course of the study which slightly changed the calf care team composition or the calf husbandry, but the selected farmers remained in their initial role for the whole duration of the project, which guaranteed that there was no impact in either their participation or consistency.

The anonymity of the interviewed farmers and their farms was ensured in compliance with German data protection legislation. The study was approved by the central ethical committee of the Freie Universität Berlin (ZEA-Nr.: 74-Z451/21). Data was stored on local servers and was only accessible by members of this project. The animal experiment application was approved on May 4th, 2023, by the Saxony State Directorate (Sächsische Landesdirektion) and registered under the number TV vG11/2023.

### 2.3 First phase: Interview with the farmers

The first interview was an online interview (video call) using a semi-structured questionnaire. The questions asked related to the management and assessment concerning calves’ health and treatment, the staff situation on the farm, including communication within the team and with the responsible veterinarian. The question form can be made available on request by the author. This first interview focused on the daily routine on the farm to get a better understanding of the farm as well. Additionally, the farmers were asked about their perception of potential problems in the calf health-related fields. We asked the farmers about the mean prevalence during the last 12 months concerning the major diseases (diarrhea, omphalitis, and respiratory problems) and to rate their satisfaction with the occurrence of these diseases on a scale from 1 to 10, with 1 being the worst and 10 the best.

### 2.4 First phase: Farm visits

Each farm was visited twice (except the one that entered the study at a later stage and which was visited once in the first phase of the study) to assess calf health following a standardized protocol.

In total, up to 58 calves were examined at each farm visit covering five age groups: 11 calves at the age from 2 to 7 days and 8 to 21 days old, respectively, as well as 12 calves at the age from 22 to 35 days and 43 to 63 days respectively, and finally 6 calves before and 6 calves after weaning, according to availability. Since the goal of this project was to assess calf health in the farms using veterinary examinations, the groups were divided by age to evaluate specific data in their optimal timeframe. The calves were selected by chance from the groups, and their age was gathered from the herd register. A clinical examination was performed as reported by Dachrodt et al. (6) that focuses on the presence/absence of common neonatal diseases [omphalitis (O), diarrhea (D), and bovine respiratory disease (BRD)] and bodily development. In short: the heart girth was measured to determine the calves’ body weight, and the hair coat was assessed for traces indicating ectoparasites and dermatomycosis, rectal temperature was determined, the navel was evaluated for signs of inflammation by palpation, lung sounds were evaluated by auscultation, the skin turgor was determined to assess the hydration status, and the disbudding sites were assessed. Additionally, fecal samples were tested for *E. coli* F5 (K99), Rotavirus, Coronavirus, and *Cryptosporidium parvum.* Blood samples were drawn to evaluate the colostral management by determination of total protein and anemia was assessed using hemoglobin in EDTA whole blood and iron in serum. Not all of the collected and analyzed health data from the calves will be presented in this article, as the focus point is on the three main diseases (D, O, BRD) and the implementation of measures. Therefore, the examinations are only described briefly.

The husbandry conditions were also assessed using a standardized form, which can be made available on request by the authors. Calf mortality risk was retrieved from herd registers.

After the first two visits, the results of the assessments were compared with the farmers’ self-perception (mean estimated prevalence and satisfaction) with the prevalence assessed by the study veterinarians for the diseases D, O, and BRD. The presence of diarrhea was defined by „liquid or soft feces,” omphalitis as inflammatory navel symptoms (thickening and/or swelling and/or pain and/or heat, excluded uncomplicated umbilical hernia), and respiratory disease as “enhanced breathing with fever but without diarrhea,” and/or “attenuated breathing “, and/or “bronchial respiration” and/or “adventitious breath sounds.” Then, we used the Calf Health Calculator benchmarking tool (33) from the PraeRi study to compare the prevalence with other German farms. Prevalence was categorized into quantiles of comparable farms (Quantile 1: 0–10% underperforming farms, quantile 2: farm is part of the 10–25%, quantile 3: 25–50%, quantile 4: 50–75%, quantile 5: 75–90% and quantile 6: best 10%) regarding the season and system (organic or conventional) related specifically to each aforementioned disease. The quantile was calculated for both visits, and the mean of these two values was then calculated per farm. The satisfaction scale, ranging from 1 to 10, given by the farmer during self-assessment, and the mean of quantiles of both visits were displayed in scatterplots. Spearman’s correlation coefficients were calculated for the satisfaction and the mean of quantiles, as well as for the farmers’ stated prevalence and the quantiles. For these analyses, R (version 4.4.1.- “Race for Your Life”), including the package “DescTools” was used. Figures were created using Microsoft Excel© or Powerpoint©.

### 2.5 Second phase: Consultation and discussion of measures

The results of both visits were summarized and sent to the farmers in a report via email. The reports presented the number of calves and their age at the date of the visit, and a summary of the data that was taken directly on site. Then, results were presented, permitting the evaluation of the colostrum supply, the prevalence of diseases (omphalitis, diarrhea, and respiratory diseases), and nutrition and housing management. A few days later, the results were presented to the farmers and their team in person by the study team using a Powerpoint© presentation. The farm veterinarians were also invited to join these consultations. This was the case for three out of nine farms. In this presentation, the results were shown in five categories: calf health, colostrum management, nutrition, housing, and communication with staff members and among staff members from the calf rearing unit. For each category, the strengths were presented first, followed by the weaknesses of the different working areas. Recommendations for improvement in the distinct area were given. In each presentation, the dairy farm was also compared to the farms analyzed in the “PraeRi” study using the aforementioned benchmarking tool (33), so that the farmers could compare their results on a national level.

At the end of the presentation, farmers were asked to reflect on the information given and on the recommendations. Subsequently, they were asked to choose up to three measures they would like to implement to improve the process of calf rearing. The veterinary research team themselves had already selected three measures from their list that they would individually recommend. Choices of farmers and veterinary research team were compared to identify overlapping measures. At the end of this process, three to five final decisions for the implementation of measures were made, and these were written on a form, given in the Supplementary material S1.

The presentation of results and the subsequent discussion on measures were tape-recorded with the consent of the farmers and their teams. One was recorded by using the software tool for webmeetings (Webex, Cisco, San José, United States) as the conversation took place online by video call. In two of the farms, the quality of the audio recording was not sufficient due to the presence of a great number of people in a poorly noise-insulated room. These two discussions were not further analyzed in regard to barriers and motivators at the initial stage. The length of the recorded conversations ranged from 35 to 62 min, with a mean time of 47 min. These conversations have been fully transcribed and anonymized to be analyzed. The transcripts of the recorded conversations were used as material to assess barriers and motivators. Therefore, each transcription was analyzed independently by two different persons in an inductive category assignment using the QCAmap online software.^1^ Clear meaning components in the text were isolated in same-intention groups. The two analyses were then compared to create a coherent/unanimous coding guideline, which permitted the identification of the principal barriers and motivators for the farmers in the process of changing their calf-rearing management.

### 2.6 Second and third phase: Implementation of measures

To record the evolution of the mind changes, farmers received a questionnaire to be completed following each meeting, after each visit from the recommendations, until the end of the project (in total 4 visits/ timepoints; Figure 2). The questionnaire used was based on a scale developed by Michels et al. to explore the use of drones in German agriculture (34). For each recommendation that was agreed upon as compelling for the farmers, farmers had the choice to mark it on a scale from 1 to 4, (1) meaning *“I will not implement the measure,”* (2) *“I am basically willing to try it out,”* (3) *“I already have concrete steps in mind as to how I can implement this”* and (4) *“I’m already implementing this measure, but…”* with space to fill out the reason why the measure is not fully implemented or not showing results.

Here the choice marked as 1 is intended to correspond to the precontemplation stage of the transtheoretical model, 2 corresponds to the contemplation stage, 3 to the preparation stage and 4 to the action and maintenance stages (Figure 1). In this study, the action and maintenance stages were firstly fused due to the short duration of the study and the inability to control the upkeep on the recommended measures. Additionally, the farmers were invited to note the reasons for their reservations on the subject, as well as their motivations on the evaluation form.

However, after the first three visits, the researchers noted that the farmers sometimes marked the measures as implemented when it was only partially the case, or when they tried it and gave up the implementation shortly after, due to a lack of a better option. Farmers sometimes remarked this in the questionnaire, or the partial implementation was observed by the study team during their work in the stables. Therefore, the questionnaire was modified for the last visit in every farm with two new options, (5) *“I’m implementing this measure partially,”* and (6) *“I tried to implement this measure but had to give it up, because….”* We therefore focused on the first (where options 5 and 6 were not yet relevant) and the last visit.

The latest version of the questionnaire can be found in the Supplementary material S2.

### 2.7 Third phase: Assessment of calves’ health

During the third phase of the project, the farms were visited three more times, one exclusively to fill out the TTM questionnaire and provide further veterinary examinations, like analyses of the climate in the barns as part of the adopted measures, and two more times to assess the calves’ health, as well as to fill out the TTM questionnaire. The examination method was the same as in the first phase to ensure comparability of the results. The changes in calves’ health and the evaluation of measures implemented will be part of another manuscript.

## 3 Results

### 3.1 Description of the participants

The participating farmers were mostly male (eight men, one woman), with an average age of 46 years (ranging from 36 to 59 years at the time of recruitment). The educational background varied from 3-year agricultural training to 5-year agricultural studies or Master of Science, with some farmers having obtained more than the aforementioned qualifications. The farmers were all well-established in their profession, with experience in the field ranging from 10 to 35 years (average 22 years of experience).

The number of animals on the farms ranged from 500 to 1,300, with an average of 785 cows. The mean mortality risk of the calves at the farm level for 8 farms (from the age of 3 days until weaning) for the first year of the study was 8.6%, ranging from 4.5 to 17.8%. The mortality risk of one farm was not calculated as the necessary data was not available.

### 3.2 Health status of the calves at the beginning of the study: comparison of the farmers’ perceptions and the assessments by the study veterinarians

**Figure 3:**
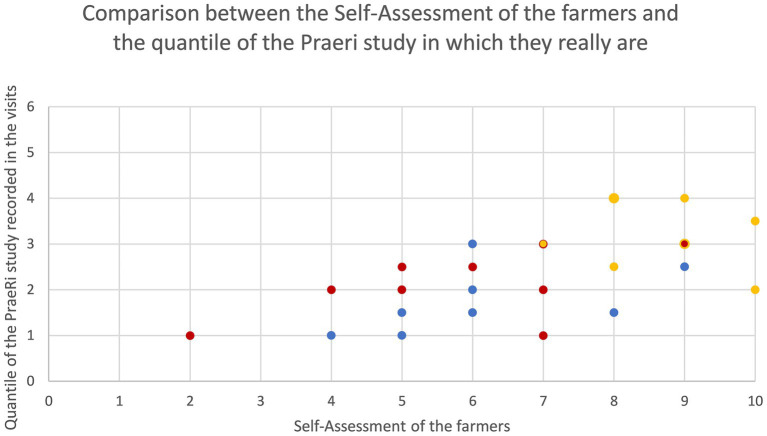
Comparison of the self-assessment of 9 farmers (1: really unsatisfied to 10: really satisfied with the current situation) and the observed prevalence by the study veterinarians categorized in quantiles based on Dachrodt et al. (33); 1: underperforming 10% of farms; 2: 75–90%; 3: 50–75%; 4: 25–50%; 5: 10–25% best farms; 6: best 10% of farms); each dot is the self-assessment of a farmer regarding each of the three main calf diseases. Red dots: diarrhea, yellow dots: omphalitis, blue dots: respiratory disease in calves. 2 points (yellow) are laying on top of another.

The farmers were mostly satisfied with the health of their calves concerning O, D, and BRD (Figure 3). There were differences in the farmers’ perception with respect to the different disease complexes: farmers were more satisfied with the incidence of O (range 7–10) and less satisfied with the incidence of D (range 2–7). The assessments by the veterinary research team were in agreement with this perception, as the prevalence of O was lower than for D and BRD. The estimates of the farmers correlated moderately to strongly with the quantiles of the PraeRi-study (Spearman correlation coefficient: 0.63 [0.82–0.33], *n* = 27), indicating that the better the farmers rated the health status, the lower the prevalence of the distinct disorder was as assessed by the veterinarians. Interestingly, the correlation between the prevalence estimated by the farmers and the quantiles based upon the prevalence of the veterinarians was slightly lower (−0.59 [95%-CI: −0.79 to −0.26]; *n* = 27) than that of the farmers’ satisfaction with the quantiles. Satisfaction and estimated prevalence of the farmers showed a high correlation (−0.75 [95%-CI: −0.88 to −0.51]; *n* = 27). However, in general, the prevalence observed indicated that farms were mostly below average concerning all three disease complexes compared to similar farms of the PraeRi-study in Germany during the same season.

Regarding assessed farm prevalence, 31.8% of the examined calves showed D (ranging from 15.5 to 53.4%), 15.4% had signs of O (prevalence ranging from 8.6 to 30.9%), and 19.0% of the examined calves showed signs of BRD (prevalence ranging from 7.3 to 39.6%).

### 3.3 Barriers and motivators

**Figure 4:**
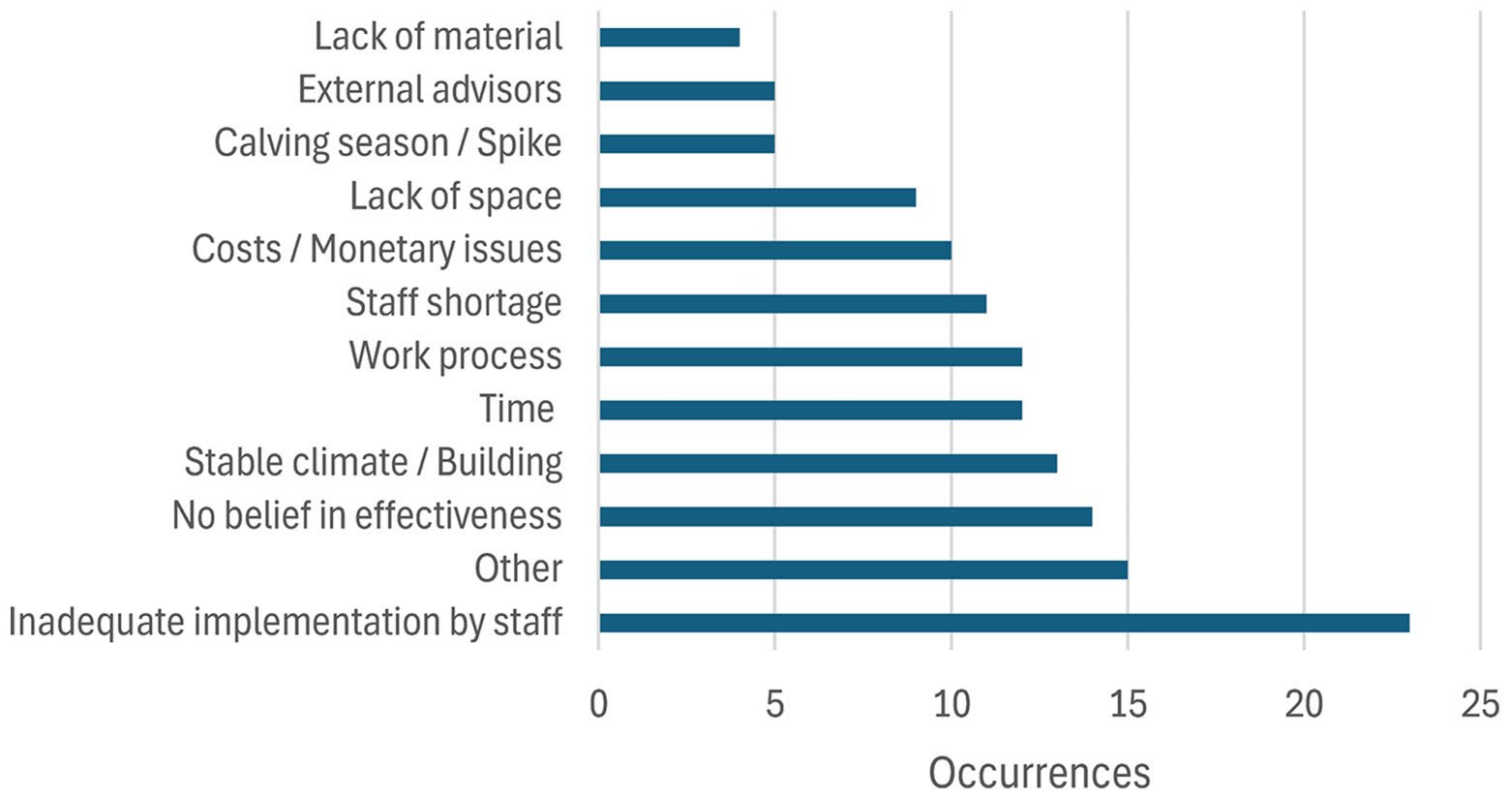
Barriers mentioned by farmers and their staff members concerning the implementation of veterinary recommendations to improve dairy calves’ health.

In total, 171 comments regarding barriers for implementation of distinct measures were evident in the transcripts of the consultations that were assorted into ten categories (Figure 4).

Category descriptions and corresponding examples can be found in the Supplementary material S3. It has to be noticed that some of the comments fell into more than one category. The first category, “Inadequate implementation by the staff,” represented 17% of the barriers that could prevent a measure from being implemented. This concerns all cases where a measure was not implemented due to insufficient knowledge, motivation, or time pressure, or even a lack of, or faulty communication between management and employees. A typical statement was “*So, to what extent I can instruct them to always try to push this through, and to what extent this will then be implemented by the employees, we will see. So, they are happy to stay in their old boots after all.*” It was noted that the second most important barrier was “Others,” due to the wide range of specific mentions made by the farmers. Unfortunately, it was not possible to fit these highly individual problematics in other well-defined categories, which shows that addressing these obstacles can sometimes necessitate a local solution more than a general political one. Some examples of these challenges would be a specific calf disease only present in one farm, the lack of motivation to drink from calves in one farm, the lack of good hay quality, the necessity to feed the calves “contaminated” milk to reach the necessary drinking volume, etc. The next biggest specific hurdle (10%) was the lack of belief in the efficiency of the measure, but also the buildings (stable layout and decay, location, and local climate). A typical quote of this category was “*They are disinfected, but this is actually a futile struggle to make the old substance more hygienic*.”

**Figure 5:**
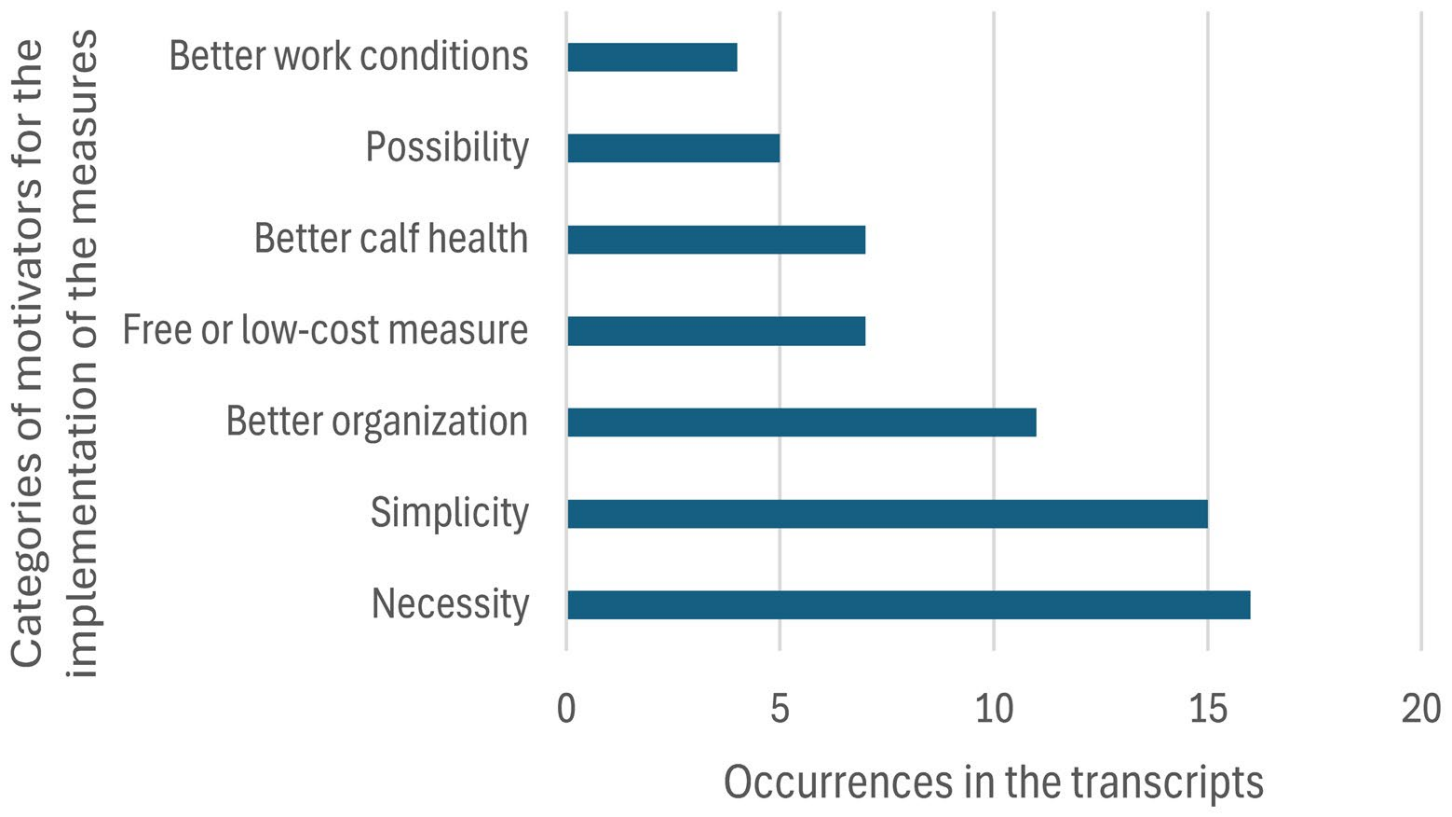
Motivators mentioned by farmers and their staff concerning the implementation of veterinary recommendations to improve dairy calves’ health.

The next barriers identified were the lack of time and the work processes in place, either 9% of comments falling in these categories, with quotes like “*The calf woman came to me herself and said, ‘We cannot do this anymore.’ We took longer than the specified time. It ended up taking around 9 h.*” Other categories, like staff shortage and costs, were not mentioned by every farmer, but were still an important topic to those who talked about them. Some participants mentioned the lack of space, often involved with the spikes of calving season, or the feed consultant or the veterinarian as a barrier to improvement (Figure 5).

The analyses of the seven recorded conversations brought up a total of 60 comments regarding motivators in the way of the measure implementation, some of them being in multiple categories. Every category description and corresponding examples for them can be found in the Supplementary material S3. The comments have been divided into seven categories. The first one, the necessity of the measure, comprises 25% of the motivators found in the conversations (“*We have to do something about the climate in the barn*”). The simplicity (“*It’s actually not particularly challenging now.*”) and the low cost of the measures also had a predominant place (37% combined). The improvement in calf health was less often mentioned directly. The mere possibility of accomplishing the measures and seeing perspectives for improvement also motivated the farmers to try to implement them (“*I’m going along with you on the disinfection, we should really try it out, maybe it will really help”*). The improvement of the working situation of staff tended to be a side benefit of the measures.

### 3.4 Management recommendations

For each specific farm, three to five final measures (median 4) were made (in total 36) and written on a form, hereby attached in the Supplementary material S1. In four out of the nine farms, the three measures retained by the farmers and the three measures given out by the study veterinarians overlapped completely. The high rate of overlapping in all 9 farms (min. 20%, max. 100%, median 67%) suggests that after the presentation, the significance and priority of the bullet points to improve were often understood and assimilated. On five farms, the study team suggested providing the calves with roughage and/or water earlier in life (see Supplementary material S1). These measures were never suggested by the farmers and were only implemented on one farm at the end of the study. Another farm tried to provide hay but gave it up as “the hay will not be touched by the calves.” The measures that were recommended are displayed in Table 1 stratified by theme. They were mostly colostrum- or feed-related (44% of the measures) but also concerned the hygiene and overall organization.

**Table 1:** Measures that were recommended to the farmers, stratified by theme, and their implementation rate.

Type of measures	Occurrences	Implemented	Implementation rate (%)
Colostrum management	8	3	38
Feeding management	8	1	13
Hygiene management	7	2	29
Medicinal measures	5	3	60
Further diagnostics	4	4	100
Housing management	2	1	50
Construction	1	0	0
Written work instructions	1	1	100
Sum	36	15	

Table 2 displays the measures stratified by the needed effort: long-term measures, like providing water or roughage from day 1, are those that need a constant behavior change. Short-term measures, on the other hand, need a single action, like building a roof or letting the veterinarians analyze the climate in the barn. Long-term measures were less likely to be implemented (fully). A reason for this circumstance, therefore, might be the mentioned barriers concerning the calf team.

**Table 2:** Measures that were recommended to the farmers, stratified by effort, and their implementation rate.

Type of measures	Not implemented	Implemented	Partially implemented	Given up	Sum
Long-term measure	10	8	5	3	26
One shot measure	2	6	2	Does not apply	9

**Figure 6:**
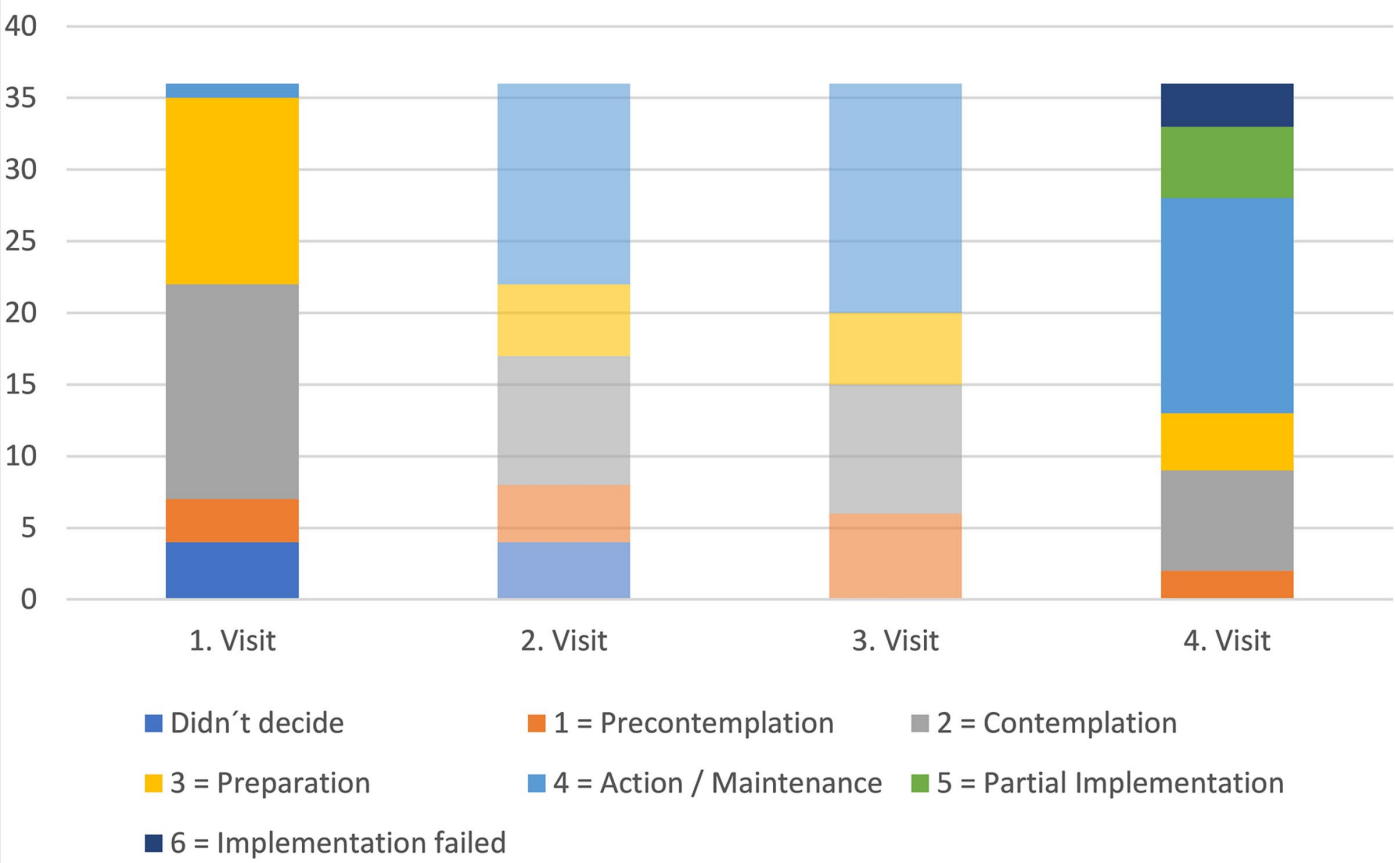
Stage of change of dairy farmers (*n* = 9) concerning measures (*n* = 36) to improve calves’ health. The 2nd and 3rd visits are lighter, as here the options “implementation failed” and “partial implementation” were not yet available.

Figure 6 displays the stage of behavior change over time. On the first visit, the measures discussed were well received, with the farmers being, for the majority of them (81%), in the contemplation, preparation, or action stage.

On the second visit, 39% of the measures were already on the action/maintenance stage. Both the contemplation and preparation stages shrank with the evolution toward the implementations, but the precontemplation stage grew slightly. Free-text answers of the farmers indicated a shift in the barriers after they first tried to implement the measures. More farmers mentioned costs as a hurdle (for the vaccines, the control of the colostrum supply or the buildings’ renovations), management problems (barrels of old disinfectant had to be used before buying new products effective against *Cryptosporidia*, or the fact that the employees did not implement the measures when the herd manager was not on the premises), or the lack of necessity for these measures (good weather so that the measure could be implemented later, not as many calves as before…).

On the third visit, almost half of the measures (46%) were on the action/maintenance stage, with measures like “written work instructions availability” being implemented according to the farmers. Free text answers of the farmers indicated that time management was still an important barrier, and that the employees did not always implement the measures according to the researchers’ recommendations: “*After the visit, the wrong work routine returns, the longer the date of the training is, the less independent the employees are. The instructions must be constantly pointed out*.”

During visits two and three, the researchers evaluated the farm regarding the measures that were agreed upon. On occasion, the farmers answered the form with a number that was considered by the researchers as “false”: e.g., the “daily re-laying of straw in the calves’ stable” was marked as implemented by a farmer, while the researchers could evaluate it as not implemented on site. For this reason, the questionnaire was changed with two more options (s. Material and Methods) that could be marked on the last visit.

On the fourth visit, the farmers tended to reevaluate their answers based on the two new options available. The number of measures ranked on the third and fourth stage of action/maintenance stayed overall the same, as the measures that worked stayed in place. Measures on the third stage (e.g., building a ceiling above the calves’ igloos) were generally measures that required more time to implement and were therefore not achievable in the relatively short time of study. The number of measures in the first two stages, however, was significantly reduced, as the farmers tried to implement them or changed their answers to the two new options: the measures were only partially implemented or were tried and failed. All the measures that were re-ranked in these two new categories had to do with the consistency in the offering of food or water, or the seriousness with which hygiene was taken. Free text answers indicated that the measures regarding the regular offering of food and water and cleaning were very dependent on the teamwork, and that the weather was a barrier to be taken into account: “*Cleaning and disinfection are implemented, occasionally poor if there is a lack of staff or frost*,” “*We give 2×4 liters per meal, feeding 3 times is technically difficult to implement as milking only takes place twice*.”

## 4 Discussion

Farmers in this study implemented a considerable proportion of recommended measures. The most important factor hindering the implementation seemed to be the lack of time of the workforce.

### 4.1 Limitations

Unlike in other qualitative studies that follow a circular or iterative research design, such an approach was not feasible in this study due to the long-term involvement with participating farms. The research process spanned more than 2 years, with pre-defined phases of data collection and farm visits, which limited the possibility to adjust the study design or interview content continuously. Nevertheless, the longitudinal design provided valuable insights into behavioral dynamics over time, which would not have been accessible through short-term or cross-sectional studies. Even for most qualitative studies, this study included only a low number of farms. We cannot assess if data saturation was achieved, as all farms were recruited, visited, and analyzed at the same time due to the long and intense study period. The aim of this part of the study, however, was to shed light on the implementation of veterinary recommendations to improve calf health in an exploratory approach. The results cannot be extrapolated to all farms and dairy systems. Indeed, the research included just large dairy farms housed in a conventional system. In smaller farms with fewer animals—even in Germany—some important barriers cited by the farmers, like the difficult organization or the too high number of simultaneous calvings, might be less relevant. The results would probably also vary within the country according to the diverse stages of modernization of the dairy industry, in which German history also plays an important role. The self-enrollment of farms to this study is also a factor that influenced the results, probably leading to a higher motivation to implement measures than an average dairy farm. The results achieved in this study could then only be extrapolated to farms, where the owner or herd manager shows a substantial interest in self-improvement. Also, the examinations and consultations performed within this study are not comparable to “normal” veterinary herd health management. The examinations that were conducted on each calf were more detailed, as the project aimed to assess comparable results to be able to offer appropriate recommendations to the farmers. Moreover, the clinical examinations of up to 58 calves lasted several hours per visit—a work that cannot be part of the daily routine of a normal veterinarian. By these intensive examinations, some of the symptoms that were brought up during the study would not have been picked up in a routine visit, or only after they reached the threshold where the illness would have been noticed by the calf team. This could have enhanced the number of calves that were assessed as sick in this study, in comparison to the perception of the farmers.

Of all the farms, only one did not implement any of the measures at the end of the study, and the eight other farms presented a very heterogeneous implementation rate, which does not allow us to draw conclusions on the impact of the farmers’ backgrounds on their level of involvement in calf health. A greater number of participants could be used in a quantitative study to link these characteristics to the attitude toward their interest in implementing new measures.

The study was also met with challenges, such as the inadequate answers on the transtheoretical questionnaire, which prevented the farmers from giving a realistic outlook on the implementation of the measures in their farms. The adaptation of the questionnaire can be regarded as beneficial, as otherwise, wrong answers would have been recorded. This questionnaire, that was initially developed by a research team that interviewed farmers in one occurrence (34) had to be adapted for our longitudinal study, where the necessity to accommodate for new answers was discovered after feedback from the participating farmers. In qualitative research, methodological flexibility—including the adaptation of questionnaires—is not a limitation, but a strength, enabling the research to remain grounded in participants’ lived experiences. Self-reports also rely on a person’s honesty or their self-assessment, which means that even with the new version of the questionnaire, it is still possible that the answers vary from the real situation. The fact that the visits were punctual and that the farm was not monitored in between does not allow a surefire way of knowing if the implementation was regular or complete.

### 4.2 Health status of the calves at the beginning of the study: comparison of the farmers’ and the study veterinarians’ assessments

The prevalences of D and BRD were higher than the median of the PraeRi-study, which was used as a reference for the comparison with other German farms. It has to be kept in mind that seasonal variations could influence the health status of calves, and as the examinations were all conducted in the cold season (from October to April), it could also mean that the BRD and D could get better, and the omphalitis prevalence could get worse in the warm season (6). Even though a significant correlation between satisfaction, self-estimated prevalence, and comparison with other farms existed, the farmers tended to assess the current state of calves’ diseases better than the assessed prevalences that the study veterinarians indicated. This phenomenon is already known concerning the percentage of lameness of adult cows (30, 35). However, to our knowledge, there is no study up to now comparing the assessment of farmers and veterinarians regarding calves’ diseases. A reason why farmers assessed the situation better than the assessment of the study veterinarians indicated might be that farmers were asked about their estimation during spring or summer, and prevalences were obtained by study veterinarians in the cold season. Moreover, as with lameness, different definitions might also exist when a calf has diarrhea, omphalitis, or BRD. It is likely that in Germany, as in the UK, veterinarians are rarely involved in day-to-day health decision-making for calves (36). This might be a reason why definitions may vary between farmers and veterinarians. A study conducted by Edwards et al. (37) showed that the level of knowledge of the veterinarians plays a big role in whether they regularly review the calf health data. Multiple factors can prevent a bigger involvement of the veterinarians in calf health: not feeling secure in the ability to efficiently direct the farmer toward better management, as well as their gender (male veterinarians were less likely to frequently review health data as their female counterparts), or even the lack of registered data (incomplete records in the farm software; 37). Further studies are needed to gain further knowledge of how farmers define and assess the health of their calves. Moreover, as concluded by Mahendran et al. (36), there might be a need for appropriate staff training and standard operating procedures.

Helping farmers realize the true prevalence of the diseases by conducting health assessments and presenting current results could also motivate them to face the issue, which they would otherwise tend to ignore, as other problems seem to be more urgent or important. According to the TTM, feedback from external sources is a driver that leads from the stage of unawareness or precontemplation to preparation (38, 39). Moreover, farmers want to be proud of their herd’s health (40) and want others to have a positive perception of them or their farm in terms of herd health (40–42). Benchmarking is therefore beneficial in terms of behavior change. While nationwide benchmarking is already in use regarding antimicrobial use (43) or udder health (44), and is available in other countries regarding calf health (45), no structural feedback is provided to German farmers on this theme. A reason, therefore, might be the poor availability of valid data.

### 4.3 Barriers and motivators

An important barrier was the farmers’ belief that their staff would not (be able to) implement the proposed measures. Farmers also mentioned that conflicts in the team or unmotivated team members hindered the implementation of measurements. Moreover, the shortage of staff and unspecific lack of time were seen as barriers. The importance of dedicated and motivated workers has been highlighted in other studies as well (46–48). In the study of Belage et al. (49), farmers mentioned a lack of compliance as one of the reasons why best milking practices cannot be followed. In the large dairy farms participating in this study, farmers could not always monitor the work of their employees on a daily basis. Another study in large dairy farms in Estonia revealed that calf care workers felt powerless and doubted their own skills to ensure the good health (50). Therefore, the knowledge, attitude, and workload of employed farm workers might be an essential key to good calf health.

Besides employees, the old buildings and the lack of space were mentioned as barriers. It should be noted that Saxony, which is situated in eastern Germany, used to be part of the German Democratic Republic. Farming structures under the Soviet regimen were more industrialized than in Western Germany. The farms built around this time period were large and are often still in use, allowing only minor changes. In the study by Palczynski et al. (47), farmers also mentioned the lack of space and the subsequent overcrowding as a barrier to overcome. These issues also made the available space harder to clean, which led to easier cross-contamination in the groups (47). Surprisingly, the farmers participating in this study did not mention financial investments or costs as often as in the other studies (42), but it was still brought up on multiple occasions as both a barrier and a motivator to implementation.

The calving spikes and lack of time, though less frequently mentioned, could also be addressed together with the findings of Vaarst and Sørensen (51), who compared farms with high- and low calf mortality. It was noted that flexibility in the workday, with time slots dedicated only to handle unexpected events and crises, was a significant factor in the management of calf health. This implementation of “flexible time” requires an initial extensive revision of existing work routines, which could demotivate the farmers, but allows for more control of the situation in the long-term.

Finally, the belief in the effectiveness of measures can be a barrier. This was especially the case when the benefit of a measure is not directly visible, e.g., the supply of colostrum of good quality or the supplementation of iron. Here, it might be helpful to monitor the outcomes to convince farmers that these measures are worth their effort.

Similarly to what has been found in our study, the perceived feasibility of the recommended measures and their effectiveness were also deemed as one of the strongest motivators in other studies (52, 53). The implementation of a measure depends on the knowledge of the person: if the farmer believes he or she possesses a sufficient amount of understanding on the theme at hand, he or she is more likely to go forward with new programs (54). This was supported by a German study that investigated the wish for and existence of written standard operating procedures (SOPs) in the calf care domain, which has also proved to be a great incentive to implement and organize new measures (55). An empathetic and involved stockperson was often named as the main driver of the calves’ good health (13, 47, 48, 56), due to their attention to detail and implication in animal health. Self-improvement was a goal, not only to be the best in a competition with other farmers (49), but also purely to achieve better calf health.

It is to be noted that considerably more barriers than motivators were mentioned in the conversations with the farmers, both in reoccurrence (133 mentions of barriers, 60 of motivators) and in variety (12 categories of barriers, 7 of motivators). As herd managers talked more frequently about barriers than motivators, motivational interviewing might be useful for veterinary consultations, as indicated by the studies of Svensson et al. (57) and Bard et al. (58). Motivational interviewing is a technique based on the TTM that promotes “change talk”: instead of convincing someone by providing arguments, the person receiving consultation is engaged to talk about changes that follow a behavior change (59). A cornerstone in this research area is the need for accurate and respectful communication between the actors of the dairy industry, as farmers are more open to changing their practices and recognizing errors, when they are addressed in a “non-condescending” way by veterinarians (1).

### 4.4 Implementation of recommendations

Herd health management and advising farmers is an important and growing part of farm animal medicine (60, 61). Nearly 20 years ago, LeBlanc et al. (60) and also Ruston et al. (62) [cited by Bard et al. (58)] concluded that it is not the knowledge itself that is lacking, but the “challenge is in effectively and consistently implementing the required management practices.” Nevertheless, to our knowledge, this is one of the first studies assessing the implementation rate of veterinary recommendations to improve calf health in dairy farms. In a research project, providing consultations to farms with high losses of calves, 25% of recommendations were implemented by the farmers (63). Compared to that study, the implementation rate observed in this study (42%) is relatively high. The mean number of recommendations per farm is comparable in both studies. One reason for the higher implementation rate might have been the way of enrollment: while the farmers self-enrolled in this study, they were contacted by the study team in the other study.

An important element of the intervention design was that farmers were not simply presented with externally imposed recommendations, but were instead encouraged to formulate their own measures for improving calf health. Writing down their intended actions probably further supported their commitment and accountability. Research has shown that involving individuals in the decision-making process and allowing them to set their own goals increases motivation and the likelihood of behavior change (64). These farmer-formulated measures were subsequently compared with the veterinarians’ professional recommendations, revealing a high degree of overlap. This indicates that farmers had a clear understanding of the key health issues and were receptive to the feedback provided by the veterinarians. It might also have been helpful for implementation if the farms had been visited beforehand. This might lead to a state of curiosity and/ or trust as the veterinarians proved to be engaged, and people had the chance to get to know each other. Especially, the farmer-veterinarian relationship is an important factor for behavior change (65).

In this study, differences were noticeable according to the feasibility of measures: those that required continuous effort, like changes in feeding routines or hygiene measures, were the least often implemented. Other recommended measures were “preparation steps” (such as stable climate analysis) to be used for further courses of action. It is partially possible that the farmers chose to implement those measures as they could be outsourced to veterinarians and did not require continuous efforts. If the results of these upstream measures were not then employed/ put into practice to improve calves’ husbandry, health, or management, their impact might not be as important. When consulting farmers, it might be helpful to sort measures according to (a) the effort needed for implementation and (b) the expected positive effect on calf health. However, more research is needed to identify both factors and strategies that increase the adoption of advice by farmers in terms of herd health.

## 5 Conclusion

This study can be seen as a small contribution to gaining a deeper understanding of veterinary consultations aiming to improve calf health in large dairy farms. Results are not directly transferable to other countries or settings. Farmers in this study had a good estimation of the health status of their calves. However, external feedback may help farmers to gain a better perception and awareness of calf health. Therefore, mortality or treatment frequency might be useful and feasible indicators for benchmarking. Moreover, veterinarians as experts in animal health are needed to provide feedback to farmers. Other studies indicated that the mere lack of awareness due to an inconsistent follow-up by the veterinarians or regular meetings to discuss the situation is the most-mentioned barrier (48, 52, 66).

Stages of change increased as around half of the measures were considered and subsequently implemented. Some measures, especially those that required the change of daily routines, were less often implemented. Also, some measures were only partially implemented or quit during the study period due to the difficulty of overhauling the existing routines. Here, evaluations by veterinarians or other external sources are needed to assess the success and efforts of the implemented measures. As many of the barriers mentioned related to the persons employed, further research is needed concerning the workforce’s motivation, dedication, and attitudes. These people, who care for the calves on a daily basis, are the ones who can easily make a change (or not).

## Data Availability

The raw data supporting the conclusions of this article will be made available by the authors, without undue reservation.

## Footnotes

1
http://www.qcamap.org

## Supplementary material

SUPPLEMENTARY MATERIAL 1 Recommended/ selected measures on the nine farms of the study.

SUPPLEMENTARY MATERIAL 2 Questionnaire for assessing the stage of change according to the Transtheoretical Model (extract for only the first measure).

SUPPLEMENTARY MATERIAL 3 Categories for barriers and motivators, their definition and examples.
