# Supplementary material for: German dairy farmers’ implementation of veterinary recommendations to improve calf health—a qualitative study based on the transtheoretical model
Source: Front Vet Sci. 2025 Dec 19;12:1695330. doi: 10.3389/fvets.2025.1695330 (PMC12758409; doi:10.3389/fvets.2025.1695330)
Supplement: SUPPLEMENTARY MATERIAL 1 — Recommended/ selected measures on the nine farms of the study. [file Supplementary_file_1.docx]

S1: Recommended/ selected measures on the nine farms of the study

| **Farm 1** | Regular colostrum quality control  Check colostrum supply regularly (blood and Brix tool)  Disinfectant on dry floors  Check barn climate + pathogen detection by veterinarians |
| --- | --- |
| **Farm 2** | Calves receive an iron injection  Navel disinfection with iodine solution and gloves  Hay and water offered from day 1  Pathogen diagnostics of respiratory diseases by veterinarians |
| **Farm 3** | Improvement in colostrum supply (target: 4 litres in the first 4 hours)  Improvement in hygiene (target: daily re-littering)  Increase in the amount of feed (2x daily 5 litres or 3x daily)  Written work instructions  Roof over calves’ igloos |
| **Farm 4** | Cleaning and disinfection measures  Repeated colostrum administration  Offering hay from day 1 |
| **Farm 5** | Increase the amount of colostrum (4 litres)  Regular colostrum quality control  Pathogen diagnostics for respiratory diseases  Vaccination against calf flu  Provide water and roughage from day 1 |
| **Farm 6** | Regular colostrum quality control  Earlier group housing (from day 21)  Water from day 1 |
| **Farm 7** | Daily littering of individual igloos  Use of cryptosporidia disinfectant  Stall climate analysis  Hay from day 1 |
| **Farm 8** | Stall climate analysis (and reduce respiratory diseases)  Maternal vaccination  Faster separation of cow and calf  Use of cryptosporidia disinfectant |
| **Farm 9** | Check / change disinfectant  Maternal vaccination  Offer hay (make canisters)  Check colostrum supply (TP/IgG) |
