# Supplementary material for: German dairy farmers’ implementation of veterinary recommendations to improve calf health—a qualitative study based on the transtheoretical model
Source: Front Vet Sci. 2025 Dec 19;12:1695330. doi: 10.3389/fvets.2025.1695330 (PMC12758409; doi:10.3389/fvets.2025.1695330)
Supplement: SUPPLEMENTARY MATERIAL 2 — Questionnaire for assessing the stage of change according to the Transtheoretical Model (extract for only the first measure). [file Supplementary_file_2.docx]

Farm:___________________ Person:_________________________ Date:______

Measure 1: ____________________________________________________

Which statement do you agree with in relation to this measure?

1. I will not implement the measure.
2. I am basically willing to try it out.
3. I already have concrete steps in mind as to how I can implement this.
4. I’m already implementing this measure, but…
5. I’m implementing this measure partially.
6. I tried to implement this measure but had to give it up, because…

What problems do you see in relation to this measure?

What do you expect from this measure?

Measure 2: ____________________________________________________

Which statement do you agree with in relation to this measure?

1. I will not implement the measure.
2. I am basically willing to try it out.
3. I already have concrete steps in mind as to how I can implement this.
4. I’m already implementing this measure, but…
5. I’m implementing this measure partially.
6. I tried to implement this measure but had to give it up, because…

What problems do you see in relation to this measure?

What do you expect from this measure?

Measure 3: ____________________________________________________

Which statement do you agree with in relation to this measure?

1. I will not implement the measure.
2. I am basically willing to try it out.
3. I already have concrete steps in mind as to how I can implement this.
4. I’m already implementing this measure, but…
5. I’m implementing this measure partially.
6. I tried to implement this measure but had to give it up, because…

What problems do you see in relation to this measure?

What do you expect from this measure?

Measure 4: ____________________________________________________

Which statement do you agree with in relation to this measure?

1. I will not implement the measure.
2. I am basically willing to try it out.
3. I already have concrete steps in mind as to how I can implement this.
4. I’m already implementing this measure, but…
5. I’m implementing this measure partially.
6. I tried to implement this measure but had to give it up, because…

What problems do you see in relation to this measure?

What do you expect from this measure?

Measure 5: ____________________________________________________

Which statement do you agree with in relation to this measure?

1. I will not implement the measure.
2. I am basically willing to try it out.
3. I already have concrete steps in mind as to how I can implement this.
4. I’m already implementing this measure, but…
5. I’m implementing this measure partially.
6. I tried to implement this measure but had to give it up, because…

What problems do you see in relation to this measure?

What do you expect from this measure?

Measure 6: ____________________________________________________

Which statement do you agree with in relation to this measure?

1. I will not implement the measure.
2. I am basically willing to try it out.
3. I already have concrete steps in mind as to how I can implement this.
4. I’m already implementing this measure, but…
5. I’m implementing this measure partially.
6. I tried to implement this measure but had to give it up, because…

What problems do you see in relation to this measure?

What do you expect from this measure?
