# Supplementary material for: German dairy farmers’ implementation of veterinary recommendations to improve calf health—a qualitative study based on the transtheoretical model
Source: Front Vet Sci. 2025 Dec 19;12:1695330. doi: 10.3389/fvets.2025.1695330 (PMC12758409; doi:10.3389/fvets.2025.1695330)
Supplement: SUPPLEMENTARY MATERIAL 3 — Categories for barriers and motivators, their definition and examples. [file Supplementary_file_3.docx]

S 3: Categories for barriers and motivators, their definition and examples

| **Barriers** | **Definition** | **Example** |
| --- | --- | --- |
| **No belief in effectiveness** | The farmer doesn’t see the necessity to implement | “I had already stopped the vaccination completely because I was afraid that it hadn't worked.” |
| **Staff shortage** | Lack of personnel to implement the measure | “You need a carer for them, that's where it gets stuck somehow.” |
| **Cost / Economical Situation of the farm** | Additional costs due to measure or not enough money to implement the measure. | “it all costs money and it shouldn't go wrong or be an experiment” |
| **Stable climate / Building structure** | Structural conditions (stable layout, location, local climate) as an obstacle to realization, also problems with frost, e.g. with igloos. | “They are disinfected, but this is actually a futile struggle to make the old substance more hygienic.” |
| **Inadequate implementation by staff** | The personnel are not able to implement the measure due to insufficient knowledge, motivation, or time pressure, or even a lack of or faulty communication between management and employees | “The question remains as to whether this can be left to the workers, it would have to be tested, whether we really say that this should be part of the routine, milking and taking samples.” |
| **Lack of material** | No disinfectant available, missing or defective equipment, or incorrect equipment; medications/agents not available | “But there are currently no scales available.” |
| **Work process** | The work process does not allow the implementation / would require major changes | “Well, he can't smell when it was born. There's not much else you can do.” |
| **Calving season / Spike** | The farm undergoes periods of time where more calves are born than usual, and it presents an obstacle to implementation | “In some months, we have already reached the limits of our capacity. There is no question that in the future we will also sell pregnant heifers to break the peaks a little. That's true, I admit it.” |
| **Lack of space** | Size of the farm as an obstacle to realization | “Then it was not possible for the staff to deal with it properly because everything was full.” |
| **External advisors** | The farm feed consultant/veterinarian are considered obstacles in the implementation | “There is always a new doctor who actually provides care; that's how things are developing. Everyone always has something new, then things change again.” |
| **Time** | The implementation would take too much time in the working day | “I'm a little unsure, firstly because I have very little time for it.” |
| **Other** | Difficulties to implement in practice for other reasons | “Well, they also said that they would have diarrhea if they got more milk.” |

| **Motivators** | **Definition** | **Example** |
| --- | --- | --- |
| **Simplicity** | Measure is quick and easy to implement | *“And you drip it onto the disc and read off the number. It's actually not particularly challenging now.”* |
| **Better organization** | Measure can be integrated into the daily workflow without additional effort and brings more efficiency (for example by combining two tasks). | *"Yes, and if you put it all down and make it ready so that you don't have to search for it and defrost it, then it's all ready."* |
| **Costs** | Measure is not associated with additional costs, or only low costs | *“such a net is also affordable”* |
| **Necessity** | Farmers consider the measure to be necessary (and have been for a long time) | *“We have to do something about the climate in the barn”* |
| **Possibility** | Farmer sees possibilities/ perspectives for improvement | *“I’m going along with you on the disinfection, we should really try it out, maybe it will really help”* |
| **Better calf health** | Farmer can see the value of the measure on the improvement of calf health | *“And that's precisely because all the calves, including the boys... Yes. That would boost their immune status.”* |
| **Better work conditions** | The measure will improve the work conditions (time, physical exhaustion, environment) for the farm workers | *"But at the end of the day, they benefit when fewer calves are ill, so they're more motivated. We've noticed that too."* |
